# Data from differentially expressed proteins in platelet components associated with adverse transfusion reactions

**DOI:** 10.1016/j.dib.2019.104013

**Published:** 2019-06-12

**Authors:** Chaker Aloui, Céline Barlier, Danielle Awounou, Stéphane Claverol, Jocelyne Fagan, Fabrice Cognasse, Olivier Garraud, Sandrine Laradi

**Affiliations:** a French Blood Bank (EFS) Auvergne-Rhône-Alpes, Saint-Etienne, France; b GIMAP-EA3064, University of Lyon, Saint-Etienne, France; c Proteome Platform, CGFB, University of Bordeaux Segalen, Bordeaux, France; d National Institute of Blood Transfusion (INTS), Paris, France

**Keywords:** Label-free nano-LC-MS/MS, Platelet proteomics, Transfusion, Adverse transfusion reactions, ATR, adverse transfusion reaction, GO, gene ontology, DE, differentially expressed, PC, platelet component, PPC, buffy-coat-derived pooled PC, SDA-PC, single donor apheresis PC, ATR-PC, PC associated with an ATR, No. ATR PC, PC not associated with an ATR (controls of ATR-PC)

## Abstract

The presented dataset was used for the study focused on the search for differentially expressed proteins in blood platelet components (PCs) associated with adverse transfusion reactions (ATRs). Pellets of ATR platelet components and their controls were subjected to high-throughput proteomics analysis using a Q Exactive high-resolution tandem mass spectrometer. The data reported here constitutes an extension of “Differential protein expression of blood platelet components associated with adverse transfusion reactions” article Aloui et al., 2018. The reported data herein have been deposited into the ProteomeXchange Consortium via the PRIDE partner repository with the dataset identifiers PXD003510 for the pooled platelet components (PPCs) and PXD008886 for the apheresis platelet components (SDA-PCs) associated with ATRs.

**Table:** Specifications table

Subject area	*Biology*
More specific subject area	*Platelet proteomics profiling associated with adverse transfusion reactions (ATRs)*
Type of data	*Mass spectrometry raw files, tables and figure*
How data was acquired	*Peptides were analysed on an Ultimate 3000 nanoLC system (Dionex, Amsterdam, The Netherlands) coupled to an Electrospray Q Exactive quadrupole Orbitrap benchtop mass spectrometer (Thermo Fisher Scientific, San Jose, CA, USA). Data were searched with SEQUEST through Proteome Discoverer 1.4 (Thermo Fisher Scientific) against the Homo sapiens reference proteome set (UniProt version 2015–07). Raw LC-MS/MS data were then imported into Progenesis QI 2.0 (Nonlinear Dynamics Ltd., Newcastle, UK) to analyse the label-free quantitative data.*
Data format	*Raw and processed data*
Experimental factors	*Platelet pellets from leukodepleted platelet components (PCs) were sampled from 6 PCs implicated in ATRs and in matched controls. The collected samples were centrifuged at* 1500 rpm *for* 10 min*. The PC pellet was washed with sterile 1X PBS. The pellet was then resuspended in* 80 μL *of PBS and* 20 μL *of 5X Laemmli buffer (with 0.5 M DTT). After denaturation at 100°C for* 3 min*, the samples were stored at -80°C until analysis.*
Experimental features	*For nanoLC-MS/MS analysis,* 10 mg *of each protein sample were solubilised in Laemmli buffer and deposited into a 10% acrylamide SDS-PAGE gel. Proteins were denatured, reduced, alkylated and digested with trypsin. The supernatant was collected, and an H*_*2*_*O/ACN/HCOOH (47.5:47.5:5) extraction solution was added onto the gel pieces for* 15 min*. Supernatants were dried in a vacuum centrifuge and resuspended in* 100 μL *of water acidified with 0.1% HCOOH. The peptide mixture generated was injected into the nano-liquid chromatography-mass spectrometry system.*
Data source location	*Proteome Platform, CGFB, University of Bordeaux Segalen, 33 076 Bordeaux, France*
Data accessibility	*Data within this article were deposited to the ProteomeXchange via the PRIDE repository with the following PRIDE identifiers: PXD003510*https://www.ebi.ac.uk/pride/archive/projects/PXD003510*(for PPCs) and PXD008886*https://www.ebi.ac.uk/pride/archive/projects/PXD008886*(for SDA-PCs).*
Related research article	*C. Aloui, C. Barlier, S. Claverol, J. Fagan, D. Awounou, E. Tavernier, D. Guyotat, H. Hamzeh-Cognasse, F. Cognasse, O. Garraud, S. Laradi, Differential protein expression of blood platelet components associated with adverse transfusion reactions, J Proteomics 194 (2018) 25–36*[1].

**Table:** 

**Value of the data** • This dataset presents the first exploration of the platelet proteomic signature associated with adverse transfusion reaction (ATRs) in two types of PCs • These data constitute an interesting resource, essential to direct subsequent/future studies, especially in the context of inflammation • These data provide a basis for subsequent comparisons of differential protein expression of blood platelet component associated with ATRs, including the investigation of the biological processes such as i) between platelet pellets and supernatants ii) between different preparation processes • This proteomics analysis paves the way to a better understanding of the pathophysiological mechanisms involved in ATRs • The provided dataset can be useful for transfusion medicine in order to improve transfusion safety and efficacy.

## 1 Data

The obtained raw proteomic data were statistically analysed (ANOVA test and absolute fold change). The processing data of PPCs and SDA-PCs have been obtained after Label Free quantification process and can be consulted in Mendeley Data (https://data.mendeley.com/datasets/p8rfnkmdtp/1).

Principal component analyses (PCA) allowed us to identify and segregate the SDA-PCs and PPCs analyses into two groups as follows: ATR-PCs *versus* no. ATR-PCs (controls), as described in detail in a previously published research manuscript [1].

We obtained two lists for differentially expressed (DE) proteins, namely ATR *versus* no. ATR, and for each type of the transfused PC, PPCs and SDA-PCs. The Supplementary Tables (1–4) of the accompanying research article [1] list the corresponding gene ontology annotations, including biological processes, molecular functions and cellular components. Below, Table 1 presents the features of the final differentially expressed proteins found to be enriched that were identified as playing a significant role in degranulation, platelet activation and/or cytoskeleton remodelling and that were also involved in the signalling integrin pathway. These relevant proteins, validated by Western blotting, may represent promising candidates to improve transfusion medicine.

**Table 1:** The DE proteins in ATRs, present a significant role in degranulation/platelet activation and/or cytoskeleton remodelling and are involved in the signalling integrin pathway in ATR-PPCs and in ATR-SDA-PCs.

	DE proteins (Gene Name [ID Uniprot])	Peptide count	Abundances (Grouped): ATR-PCs	Abundances (Grouped): no.ATR-PCs	Confidence score	P-value	Ratio
In ATR-PPCs	Platelet basic protein (PPBP [P02775])	6	244 050 285	109 065 780	29.57	0.0012	2.24
	Rho-related GTP-binding protein RhoG (RHOG [P84095])	5	6 404 276	1801 718	10.89	0.0048	3.55
	Alpha-1-antichymotrypsin (SERPINA3 [P01011])	3	0	735 679	7.98	0.0000002	∼0
In ATR-SDA-PCs	Alpha-actinin-1 (ACTN1 [P12814])	685	3 894 757 912	2 081 306 441	2425.56	0.0165	1.87
	Multimerin-1 (MMRN1 [Q13201])	299	13 616 389 948	8 630 563 950	1023.55	0.0173	1.58

(ATR, adverse transfusion reactions; DE: differentially expressed; FC, Fold change; PPC, buffy-coat-derived pooled PCs; SDA-PCs, single donor apheresis PCs)

## 2 Experimental design, materials and methods

**Fig. 1:**
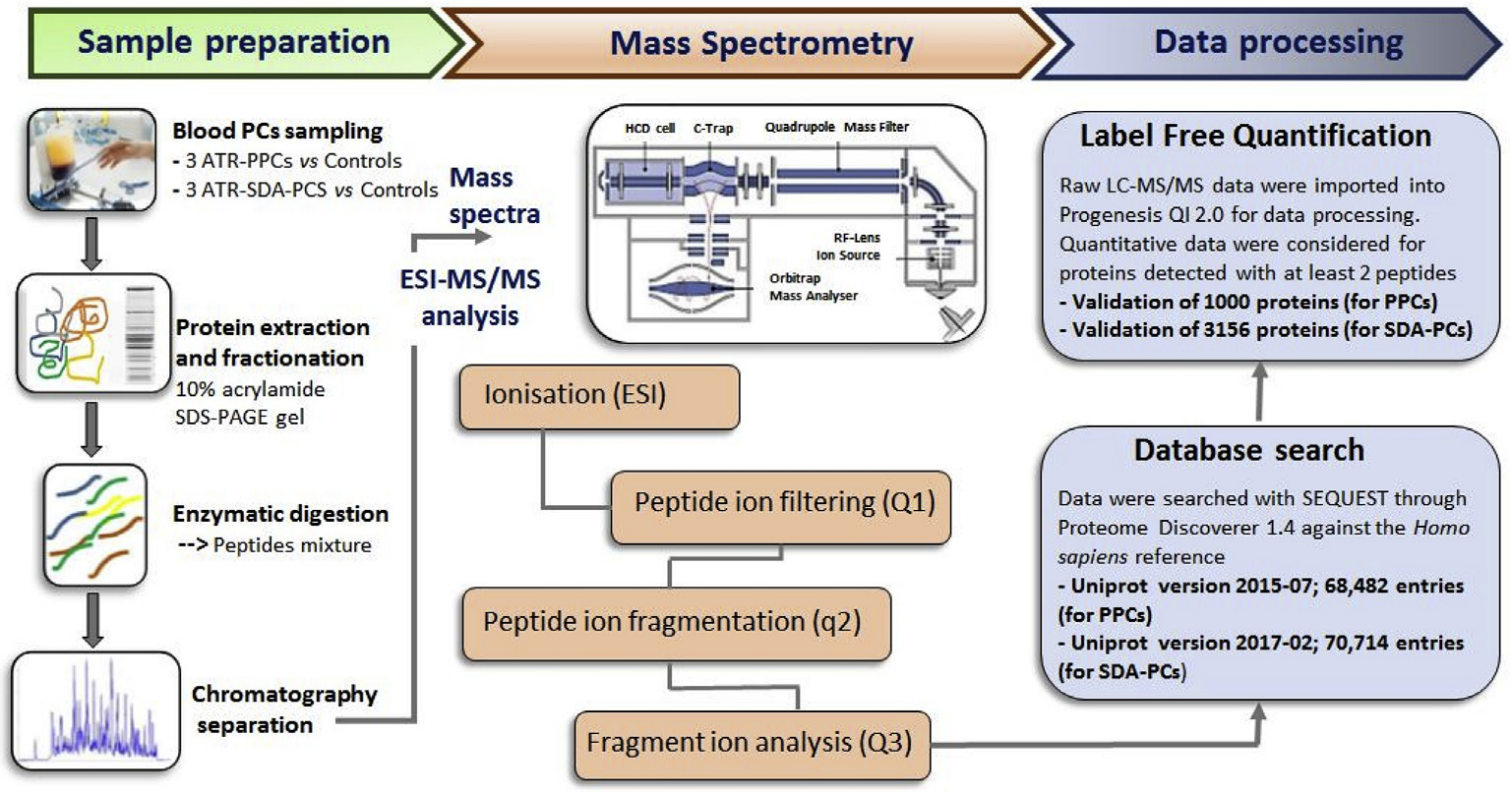
Flowchart of experiments using the label-free method (nano-LC-ESI Orbitrap Q Exactive mass spectrometer) and data processing. (ATR, adverse transfusion reaction; ESI, electrospray ionisation; LC, liquid chromatography; PC, platelet component; PPC, buffy-coat-derived pooled PCs; SDA-PCs, single donor apheresis PCs).

Sample collection and preparation, mass spectrometry and data analysis are summarised in Fig. 1. The results of the processed data are formatted in Excel (.xlsx) tables in Mendeley Data (https://data.mendeley.com/datasets/p8rfnkmdtp/1).

### 2.1 Sample collection and inclusion criteria

This study was approved by the Ethics Committee of the University Hospital of Saint-Etienne (ID RCB 2014-A00405–42). This work focused on febrile non-haemolytic reactions (FNHRs) with a severity score of 2–3 and an imputability score of 2–3 according to the NOTIFY Library definition [2]. Leftovers from the incriminated PC bags were shipped immediately to the research facilities for handling. Six PCs associated with an ATR (ATR-PC) were collected (three ATR-SDA-PCs and three ATR-PPCs), analysed and compared to six matched controls.

The collected samples were immediately prepared as described [1], and the samples were stored at -80°C.

### 2.2 Sample preparation for label-free analysis

Ten micrograms of each protein sample were solubilised in Laemmli buffer and loaded onto a 10% acrylamide SDS-PAGE gel. After colloidal blue staining, protein profiles were cut into 4 bands, and each band was cut again into 1 mm × 1 mm gel pieces. Gel pieces were destained in 25 mM ammonium bicarbonate in 50% ACN, rinsed twice in ultrapure water and shrunk in ACN for 10 min. After removing the ACN, the gel pieces were dried at room temperature, covered with a trypsin solution (10 ng/μL in 40 mM NH_4_HCO_3_ and 10% ACN), rehydrated at 4°C for 10 min, and finally incubated overnight at 37°C. Gel pieces were then incubated for 15 min in 40 mM NH_4_HCO_3_ and 10% ACN at room temperature. The supernatant was collected, and an H_2_O/ACN/HCOOH (47.5:47.5:5) extraction solution was added onto the gel pieces for 15 min. The extraction step was repeated twice. Supernatants were dried in a vacuum centrifuge and resuspended in 100 μL of water acidified with 0.1% HCOOH. Samples were stored at -20°C.

### 2.3 nLC-MS/MS analysis

The peptide mixture was analysed on an Ultimate 3000 nanoLC system (Dionex, Amsterdam, The Netherlands) coupled to an Electrospray Q Exactive quadrupole Orbitrap benchtop mass spectrometer (Thermo Fisher Scientific, San Jose, CA, USA), as previously described [1]. Data were acquired using the Xcalibur 2.2 software in a data-dependent mode. MS scans (m/z 300–2000) were recorded at a resolution of R = 70 000 (@ m/z 200) and an AGC target of 1 x 10^6^ ions collected within 100 ms. The dynamic exclusion was set to 30 s, and the top 15 ions were selected from the fragmentation in HCD mode. MS/MS scans with a target value of 1 x 10^5^ ions were collected with a maximum fill time of 120 ms and a resolution of R = 35 000. Additionally, only + 2 and + 3 charged ions were selected for fragmentation. The other settings were as follows: (i) neither sheath nor auxiliary gas flow; (ii) heated capillary temperature of 260°C; (iii) normalised HCD collision energy of 25% and (iv) an isolation width of 3 m/z.

### 2.4 Database search and results processing

Data were searched with SEQUEST through Proteome Discoverer 1.4 (Thermo Fisher Scientific) against the *Homo sapiens* reference proteome set (UniProt version 2015–07; 68 482 entries). Spectra from peptides higher than 5000 Da or lower than 350 Da were rejected. The mass accuracy of the monoisotopic peptide precursor and peptide fragments was set to 10 ppm and 0.02 Da, respectively; only b- and y-ions were considered for mass calculation. Oxidation of methionines ( + 16 Da), propionamide ( + 71 Da) and carbamidomethylation of cysteines ( + 57 Da) were considered as variable modifications. Two missed trypsin cleavages were allowed. Peptide validation was performed using the Percolator algorithm [3], and only “high confidence” peptides were retained, corresponding to a 1% false positive rate at the peptide level.

### 2.5 Label-free quantitative data analysis

Raw LC-MS/MS data were imported into Progenesis QI 2.0 (Nonlinear Dynamics Ltd, Newcastle, UK). Data processing was as follows: detect features, align features across the samples, calculate volume integration for 2–6 charge-state ions, normalise the ratio median, import the sequence information, use ANOVA test at the peptide level to filter features at p < 0.05, calculate the protein abundance (sum of the volume of corresponding peptides), and finally perform ANOVA test at the protein level to filter features at p < 0.05. Only non-conflicting features and unique peptides were considered for the calculation at the protein level. Quantitative data were considered for proteins quantified by a minimum of 2 peptides. The processing data of PPCs and SDA-PCs pellets were respectively presented in Mendeley Data (https://data.mendeley.com/datasets/p8rfnkmdtp/1).”

### 2.6 Functional analysis: bioinformatics and data interpretation

Differentially expressed proteins (ANOVA p-value < 0.05 and absolute fold change (|FC| > 1.5) were used for the principal component analysis, the biological clustering and the interpretation of results. The Search Tool for the Retrieval of INteracting Genes/proteins (STRING) database (version 10.5) (http://string-db.org) [4] was used to establish the protein-protein interaction network of the significantly DE proteins. To examine the biological significance of the DE proteins, we performed GeneCodis3 [5] to annotate them for the gene ontology (GO) terms enrichment as follows: biological processes (BP), molecular functions (MF) and cellular components (CC). An enrichment analysis was performed with the Kyoto Encyclopedia of Genes and Genomes (KEGG) version 82.0 (http://www.genome.jp/kegg/pathway.html) [6] to investigate the metabolic pathway implications of the DE proteins. The signalling pathways were analysed by the Ingenuity Pathway Analysis platform (release 2017–09–14) (IPA®, QIAGEN, Redwood City, CA, USA, www.qiagen.com/ingenuity). Interactive pathways were generated to observe potential direct and indirect interactions among the differentially regulated proteins and to highlight the top canonical pathways, predicted diseases and biological functions associated with the DE proteins.
